# The most dangerous hospital or the most dangerous equation?

**DOI:** 10.1186/1472-6963-7-185

**Published:** 2007-11-15

**Authors:** Yu-Kang Tu, Mark S Gilthorpe

**Affiliations:** 1 Biostatistics Unit, Centre for Epidemiology and Biostatistics, University of Leeds, 30/32 Hyde Terrace, Leeds, LS2 9LN, UK; 2 Leeds Dental Institute, University of Leeds, Clarendon Way, Leeds, LS2 9LU, UK

## Abstract

**Background:**

Hospital mortality rates are one of the most frequently selected indicators for measuring the performance of NHS Trusts. A recent article in a national newspaper named the hospital with the highest or lowest mortality in the 2005/6 financial year; a report by the organization Dr Foster Intelligence provided information with regard to the performance of all NHS Trusts in England.

**Methods:**

Basic statistical theory and computer simulations were used to explore the relationship between the variations in the performance of NHS Trusts and the sizes of the Trusts. Data of hospital standardised mortality ratio (HSMR) of 152 English NHS Trusts for 2005/6 were re-analysed.

**Results:**

A close examination of the information reveals a pattern which is consistent with a statistical phenomenon, discovered by the French mathematician de Moivre nearly 300 years ago, described in every introductory statistics textbook: namely that variation in performance indicators is expected to be greater in small Trusts and smaller in large Trusts. From a statistical viewpoint, the number of deaths in a hospital is not in proportion to the size of the hospital, but is proportional to the square root of its size. Therefore, it is not surprising to note that small hospitals are more likely to occur at the top and the bottom of league tables, whilst mortality rates are independent of hospital sizes.

**Conclusion:**

This statistical phenomenon needs to be taken into account in the comparison of hospital Trusts performance, especially with regard to policy decisions.

## Mortality in NHS hospitals

According to an article in the Daily Telegraph [1] (accessed online on 25/04/2007), the George Elliot Hospital (the only hospital run by the George Elliot Hospital NHS Trust) may have been the most dangerous hospital in England during the 2005/6 financial year. This is because its Hospital Standardised Mortality Ratio (HSMR) was 1.43, i.e. the number of patient deaths in this hospital was 43% higher than expected. In contrast, the hospital run by the Royal Free Hampstead Trust may have been the safest, since its HSMR was only 0.74, i.e. the number of patient deaths in this Trust was 26% lower than expected. The source of information in the Daily Telegraph was provided by an organization called Dr Foster Intelligence, which recently published a report entitled "How healthy is your hospital" [2], in which the performance of NHS Trusts was assessed against several indicators, such as post-operative mortality and emergency readmission.

According to the Daily Telegraph, the George Elliot Hospital had problems in the areas of both finance and hospital infection, though the Royal Free Hospital seems to have its own problems too. Whilst we do not have any explanations for the higher than average mortality rate in the George Elliot Hospital NHS Trust, we know that it is a relatively small hospital with only 352 beds, and admissions totalled 42,577 during 2005/6, according to Hospital Episode Statistics [3]. Even the Royal Free Hampstead Trust is not very large, with around 900 beds in current use for patient care, with total admissions of 62,062 during 2005/6 [3]. In contrast, the Leeds Teaching Hospitals NHS Trust has three hospitals and 2,370 beds (according to the Daily Telegraph website) with a total of 190,604 admissions during 2005/6 [3]. There is clearly huge variation in the sizes of Trusts and hence the number of patients they treat, and the question we consider is does size matter? We shall explain in this article why, from a statistical viewpoint, the size of a hospital may be a crucial factor as to whether or not that hospital appears at the top or the bottom of any league table.

## Why size matters

First let us use a simple example to illustrate why the size of a hospital can matter. Suppose hospitals in England have only five different sizes – 200, 400, 600, 800 and 1000 beds – and they undertake 100, 200, 300, 400, and 500 coronary artery bypass graft operations each year, respectively. Also suppose that the post-operative mortality rate is nominally 10%, irrespective of hospital size. The expected number of deaths for the different sized hospitals should then be 10, 20, 30, 40, and 50, respectively. Nevertheless, it is inevitable that across the years there will be some variation; for instance, in some hospitals with 200 beds only 8 patients may die, whilst in other hospitals of the same size 12 patients may die. The overall average mortality rate nevertheless remains 10%. Suppose the extent of variation (standard deviation) between hospitals of the same size is similar across all hospitals, e.g. the observed number of deaths plus or minus its standard deviation is 10 ± 3, 20 ± 3, 30 ± 3, 40 ± 3, and 50 ± 3, respectively. So what of the observed mortality rates? From the smallest to largest hospital, the observed mortality rates have 95% confidence intervals of 4.0%–16.0%, 7.0%–13.0%, 8.0%–12.0%, 8.5%–11.5%, and 8.8%–11.2%, respectively. If we were to make a league table for these hospitals, the smaller hospitals are more likely to be found at the bottom *and *the top the league table. Nevertheless, if factors related to the success of coronary artery bypass surgery act in a similar way across different sized hospitals, then variations in the number of deaths for larger hospitals would be expected to be greater than for smaller hospitals.

Many factors affect the performance indicators of hospitals, such as the post-operative mortality rate. There has been a continuing debate regarding whether or not these indicators can really measure the quality of healthcare provided by a hospital Trust [4-9]. Any hospital that treats more patients with higher risks or greater complexity may show higher mortality rates. However, notwithstanding the controversy regarding the validity of performance indicators, it is important to note that the extent of variation in the number of deaths in hospitals of the same size is *not *in proportion to the size of the hospital, but is in proportion to the square root of its size [10,11]. Therefore, for our simple example, if all the factors related to post-operative mortality (e.g. case-mix, staff experiences, and support from post-operative care units, etc.) were comparable for all hospitals and operated in similar ways across hospitals of different sizes, the variation of the observed number of deaths would be 10 ± 3.0, 20 ± 4.2, 30 ± 5.2, 40 ± 6.0, and 50 ± 6.7, rather than 10 ± 3.0, 20 ± 6.0, 30 ± 9.0, 40 ± 12.0, and 50 ± 15.0. The observed mortality rates would then have 95% confidence intervals of 4.0%–16.0%, 5.8%–14.2%, 6.5%–13.5%, 7.0%–13.0%, and 7.3%–12.7%, respectively. Hence, the smaller hospitals are still more likely to be found at the bottom and the top the league table.

From a statistical viewpoint, this is because the standard deviation of the sampling distribution of the mean, i.e. the standard error of the mean, is inversely related to the square root of the sample size: σx¯=σ/n
 MathType@MTEF@5@5@+=feaafiart1ev1aaatCvAUfKttLearuWrP9MDH5MBPbIqV92AaeXatLxBI9gBaebbnrfifHhDYfgasaacPC6xNi=xH8viVGI8Gi=hEeeu0xXdbba9frFj0xb9qqpG0dXdb9aspeI8k8fiI+fsY=rqGqVepae9pg0db9vqaiVgFr0xfr=xfr=xc9adbaqaaeGacaGaaiaabeqaaeqabiWaaaGcbaacciGae83Wdm3aaSbaaSqaaiqbdIha4zaaraaabeaakiabg2da9maalyaabaGae83WdmhabaWaaOaaaeaacqWGUbGBaSqabaaaaaaa@33BE@. This equation appears in every introductory statistics textbook and was first stated by the French mathematician de Moivre in 1730. This equation shows that the greater the sample size, the less likely is the sample mean to fluctuate, i.e. the variation is much greater for small hospitals and much less for large hospitals.

It has been noted in the literature that there is "over-dispersion" of performance indicators for smaller hospitals or Primary Care Trusts [7], and therefore the use of league tables for the ranking of hospital performance may be misleading [4,5,7,12]. Quality control charts [4,8] and funnel plots [5,7,12,13] have been proposed as alternative strategies to compare hospital performance, and to identify those for whom performance is below the national standard. To understand why quality control charts and funnel plots are more appropriate methods for comparing the performance of hospitals, it is crucial for health services researchers, doctors, and patients to appreciate fully the significance of de Moivre's equation.

## Throwing the die of death

Suppose there is an imaginary fair die with 20 surfaces. One surface of the die is black and the other 19 are white. When the die is thrown, the probability of the black surface showing is 0.05, i.e. when the die is thrown 20 times, we expect on average to see the black surface only once. However, due to the nature of all random processes, in each round of 20 throws the black surface may or may not show, or might show more than once. Similarly, although the black surface is expected to show 50 times when the die is thrown 1,000 times, the black surface may actually show more or less than 50 times. From a statistical viewpoint, an experiment like this is known as a Bernoulli trial [14,15]. The results collected from performing multiple independent Bernoulli trials, such as throwing our twenty-sided die 1,000 times, follow a binomial distribution [14,15], which can be used to calculate the variation in the number of times the black surface is expected to show. By denoting the number of throws (trials) as *n *= 1,000 and the probability of obtaining black as *π *= 0.05, statistical theory tells us that the population mean, *nπ*, is 50 and the standard error of this mean, nπ(1−π)
 MathType@MTEF@5@5@+=feaafiart1ev1aaatCvAUfKttLearuWrP9MDH5MBPbIqV92AaeXatLxBI9gBaebbnrfifHhDYfgasaacPC6xNi=xH8viVGI8Gi=hEeeu0xXdbba9frFj0xb9qqpG0dXdb9aspeI8k8fiI+fsY=rqGqVepae9pg0db9vqaiVgFr0xfr=xfr=xc9adbaqaaeGacaGaaiaabeqaaeqabiWaaaGcbaWaaOaaaeaacqWGUbGBiiGacqWFapaCdaqadaqaaiabigdaXiabgkHiTiab=b8aWbGaayjkaiaawMcaaaWcbeaaaaa@3435@, is 6.9 [10]. Consequently, the number of times the black shows has a 95% confidence interval of 36 to 64.

In 2002–2003, the average mortality following selected surgical procedures in English NHS hospital Trusts was around 5% [16]. Now suppose the die represents the probability of death following these selected surgical procedures and *n *is number of surgeries undertaken by the hospital. The 95% confidence interval for the number of deaths is between 36 and 64, i.e. the mortality rate has a 95% confidence interval between 3.6% and 6.4%. For NHS hospital Trusts undertaking 4,000 surgeries, the expected number of deaths is 200 and the standard error of the mean is 13.8, which is twice as large as that for hospital Trusts undertaking only 1,000 surgeries. However, the 95% confidence interval for the mortality rate of this larger hospital Trust is 4.3% to 5.7%, which is narrower than that for the hospital Trust undertaking only 1,000 surgeries.

**Figure 1:**
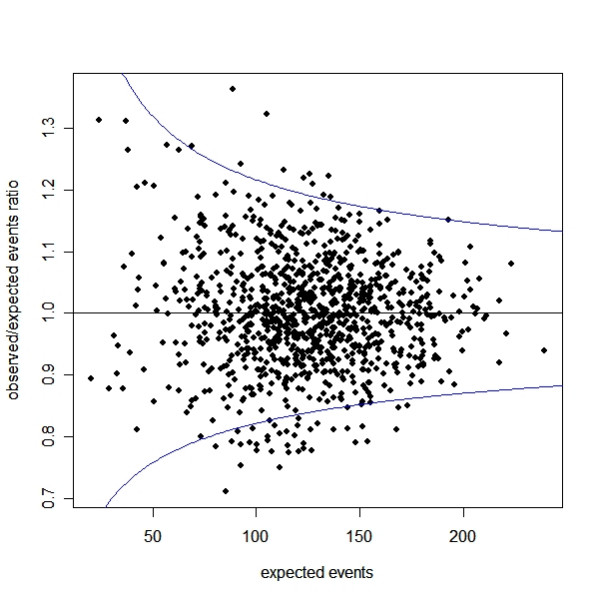
A funnel plot of the relationship between the expected number of events and the ratio of observed to expected number of events in the simulated dataset of 1,000 hospitals. The blue lines (top and the bottom of the panel) represent respectively the upper and lower 95% confidence limits of the observed/expected ratio.

A funnel plot in Figure 1 shows a simulated dataset of 1,000 hospitals in which the number of surgical procedures (Y) in each year has a mean of 2,500 and a standard deviation (SD) of 700. The mortality rate is assumed to be 5% across all sizes of hospital, so the number of expected deaths (X) has a mean of 125 and SD of 35. A random variable with zero mean and SD proportional to the square root of X is simulated to represent the variation/fluctuation in the observed mean number of deaths, and this is added to X. The vertical axis in Figure 1 is the ratio of observed number of deaths (Z) over the expected number of deaths (X), and the horizontal axis is X. If we fit a linear regression model to the data, the regression slope will be close to zero, indicating that the observed to expected ratio is independent of the expected number of deaths (i.e. hospital size), yet variation in the 95% confidence interval of these ratios (represented by the blue lines both top and the bottom of the figure) is inversely related to hospital size. Although this simulation assumes no relationship between mortality and the number of surgeries undertaken, a few hospitals are below the lower confidence limit or above the upper confidence limit, as would be expected due to chance alone 5% of the time, indicating that their performance is either alarmingly poor or extremely good. We would therefore still need to be cautious in identifying the poor or good performers using funnel plots or quality control charts, given that chance is involved. In the report published in the "How healthy is your hospital?" readers can find that the report's graphs follow a very similar pattern [2].

## The most dangerous equation

In a recently published article [10], Howard Wainer nominated de Moivre's equation as "the most dangerous equation", since being ignorant of its consequences may cost us dearly, especially with regard to policy decisions. One example given by Wainer was the project to convert large schools to many small schools simply because, amongst the high-performance schools, there seemed to be an unrepresentatively large proportion of small schools. The Bill and Melinda Gates foundation had given approximately $1.7 billion for this project, which achieved disappointing results [10]. Maybe it should be called the most expensive equation? If someone in the foundation had noted that small schools were also over-presented in the poor-performance schools, this $1.7 billion could have been saved for other purposes.

**Figure 2:**
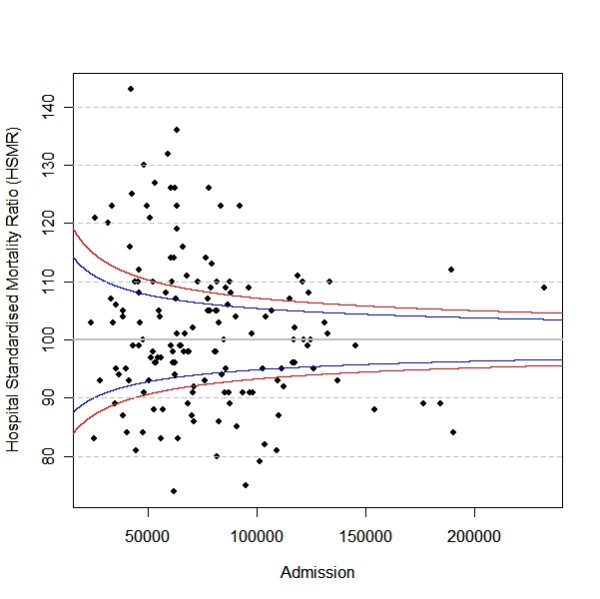
The relationship between the number of admissions and Hospital Standardised Mortality Ratio (HSMR). The variation in HSMR is inversely related to the number of admissions. The blue lines (top and the bottom of the panel) represent respectively the upper and lower 95% confidence limits of the HSMRs. The red lines (top and the bottom of the panel) represent respectively the upper and lower 99% confidence limits of the HSMRs.

Returning to hospital performance league tables, Table 1 shows the best and poorest performing NHS hospital Trusts, amongst the 152 English NHS Trusts, with respect to the hospital standardised mortality ratio (HSMR) for 2005/6, provided by "How healthy is your hospital" [2]. The number of hospital admissions within each hospital Trust during 2005/6 is used as a proxy variable for the volume of patients treated within each hospital Trust [3]. The range of admissions was between 24,269 and 232,033. Sixty-nine of the 152 (45.4%) Trusts had fewer than 65,000 admissions and 13 of them appear in Table 1; none of the 27 Trusts with admissions greater than or equal to 110,000 appears in Table 1. We may conclude that there is an unrepresentatively large proportion of small Trusts in both the best and poorest performing groups. Figure 2 shows the relationship between HSMR [2] and the number of hospital admissions [3] for the 152 English NHS hospital Trusts listed in "How healthy is your hospital" [2]. It is noted that there is less variation amongst mortality rates the greater the number of admissions, as predicted by de Moivre's equation, and this explains, in part, why many hospital Trusts in the league table (Table 1) are small ones. Figure 2 also shows that the HSMR of many NHS hospital Trusts is outside the 95% or even 99% confidence interval. Although the HSMRs of several large-sized NHS Trusts are relatively lower than those of many small Trusts, the HSMRs of these large-sized NHS Trusts are still above the upper 99% CI. However, although this may indicate the performance of these large NHS hospital Trusts is also below the bar, these Trusts would not be identified by the league table.

**Table 1:** Best and poor performing NHS Trusts based on hospital standardised mortality ratios for 2005/6 according to "How healthy is your hospital"; the information pertaining to the number of admissions in 2005/6 is obtained through the website of Hospital Episode Statistics (accessed on 21/07/2007).

**Best performing NHS Trusts**	**Number of admissions**	**Poorest performing NHS Trusts**	**Number of admissions**
Royal Free Hampstead NHS Trust	62,062	University Hospitals Coventry and Warwickshire NHS Trust	92,222
The Hammersmith Hospitals NHS Trust	95,026	Tameside and Glossop Acute Services NHS Trust	43,097
Bradford Teaching Hospitals NHS Foundation Trust	101,540	Basildon and Thurrock University Hospitals NHS Foundation Trust	62,418
St George's Healthcare NHS Trust	82,043	Dudley Group of Hospitals NHS Trust	78,036
Cambridge University Hospitals NHS Foundation Trust	109,204	The Medway NHS Trust	60,698
Homerton University Hospital NHS Foundation Trust	44,652	Mid Staffordshire General Hospitals NHS Trust	53,312
Guy's and St Thomas' NHS Foundation Trust	103,627	Burton Hospitals NHS Trust	48,324
St Mary's NHS Trust	56,244	Good Hope Hospital NHS Trust	59,487
The Royal Liverpool and Broadgreen University Hospitals NHS Trust	64,135	Kettering General Hospital NHS Trust	63,558
Weston Area Health NHS Trust	25,434	George Eliot Hospital NHS Trust	42,577

Why the George Elliot Hospital Trust had the highest mortality rate in the England in 2005/6 merits further explanation, but it might be too early to call it the *most dangerous *hospital based simply on its HSMR. As long as it remains small, it is more likely than larger hospital Trusts to be at the bottom or the top of any league table, even if the quality of care provided by the George Elliot Hospital Trust is no worse than other Trusts [9]. This is not to imply that only the size of a Trust plays a role in the random processes of variation; some studies have suggested that there exists an inverse association between hospital (surgeon) volume and surgical mortality [17,18]. In addition, there may be other important managerial and financial factors contributing to the poorest (or best) performance of some (small) hospitals. Nevertheless, using league tables to rank the performance of hospital Trusts could be potentially misleading, and there are alternative, more appropriate methods available [4-8,12,13]. Therefore, the next time we hear about the 'most dangerous/safest' city, the 'most dangerous/safest' postcode, the 'best/poorest' school etc., it is better to think again about de Moivre's equation.

## Competing interests

The author(s) declare that they have no competing interests.

## Authors' contributions

YKT conceived the ideas of this study, obtained and analysed the data, and wrote the first draft. MSG contributed to the discussion of the statistical analyses and revisions of the draft. Both authors read and approved the final manuscript.

## Pre-publication history

The pre-publication history for this paper can be accessed here:


